# Clinical factors associated with initial *Helicobacter pylori* eradication therapy: a retrospective study in China

**DOI:** 10.1038/s41598-020-72400-0

**Published:** 2020-09-21

**Authors:** Yanbo Tang, Guodu Tang, Liying Pan, Hua Zhu, Shanmei Zhou, Zhaoyong Wei

**Affiliations:** 1 Department of Gastroenterology, The First Affiliated Hospital of Guangxi Medical University, Nanning, 530021 Guangxi Province China; 2 Department of Gastroenterology, The First Affiliated Hospital of Guangxi University of Science and Technology, Liuzhou, 545002 Guangxi Province China

**Keywords:** Gastroenterology, Health care, Medical research, Risk factors

## Abstract

The eradication rate of *Helicobacter pylori* (*H. pylori*) has been decreasing every year, mainly due to the increase in antibiotic resistance. In fact, many other factors may affect *H. pylori* eradication. To analyze the clinical factors affecting the initial eradication therapy in Chinese patients with *H. pylori* infection. We conducted a retrospective study on 264 outpatients who were diagnosed with *H. pylori*-associated chronic gastritis and peptic ulcer disease between January and December 2015 at a large tertiary hospital in China. The patients were divided into three groups: ECA, RCA, and RCM (R: 20 mg rabeprazole, E: 40 mg esomeprazole, C: 0.5 g clarithromycin, A: 1.0 g amoxicillin and M: 0.4 g metronidazole). The patients were treated for 14 days and followed up for 1 year. The 14C-urea breath test (14C-UBT) was performed 4 weeks after the completion of the eradication therapy. The eradication rate was higher in ≥ 40-year-old patients than in < 40-year-old-patients (85.7% vs. 54.7%, *p* = 0.002). Multivariate analyses revealed only age ≥ 40 years to be significantly associated with a high *H. pylori* eradication rate [odds ratio (OR) 4.58, *p* = 0.003]. The *H. pylori* eradication rate in patients with duodenal ulcers was significantly higher than that in patients with gastric ulcers (79% vs. 60%, *p* = 0.012). Age could be a predictor of successful *H. pylori* eradication. Patients with duodenal ulcers had a higher *H. pylori* eradication rate than those with other lesions.

## Introduction

More than 50% of the global population has been estimated to be infected with *Helicobacter pylori* (*H. pylori*). Socioeconomic conditions and ethnicity influence *H. pylori* infection^1–3^.

The *H. pylori* infection rate is relatively higher in China, and the infection is more frequently observed in the countryside than in urban areas.

*H. pylori* is the primary cause in most cases of peptic ulcer disease (PUD), gastric cancer, and gastric mucosa-associated lymphoid tissue lymphoma (MALT). It has also been reported to cause functional dyspepsia, iron deficiency anemia, and neurodegenerative disease^4^.

Successful *H. pylori* eradication therapy can usually prevent the relapse of PUD and reduce corresponding complications even after stopping all treatments. In a previous study in which early gastric cancer was treated endoscopically, the risk of metachronous gastric neoplasms was found to decrease after *H. pylori* eradication^5^. *H. pylori* eradication could significantly decrease the risk of gastric cancer and prevent its occurrence, especially in patients with early-stage *H. pylori* infection^6,7^.

The eradication rates of *H. pylori* have been decreasing every year. Several studies have shown the eradication rates to be 75%^5,8^. Resistances to antibiotics, particularly clarithromycin, is the crucial factor affecting *H. pylori* eradication therapy^9,10^. The clarithromycin resistance rate has been found to be up to 50% in China^11^. There are regional differences for resistance rate to clarithromycin. Another literature reported that the resistance rate to clarithromycin was 17.76% in Jiaxing City of China^12^. However, *H. pylori* culture is time-consuming, difficult, and expensive and has a low positive rate. To our knowledge, few studies have been conducted on the clinical factors affecting the eradication therapy in China. We aimed to analyze the clinical factors associated with the initial eradication therapy and to explore possible solutions.

## Materials and methods

### Patients

In total, 264 outpatients aged 18–70 years with current *H. pylori*-associated chronic gastritis and PUD diagnosed between January and December 2015 were chosen for the present study. The majority of the patients were from rural areas.

The exclusion criteria were as follows:

1. A history of gastrectomy.
2. The presence of gastric cancer.
3. Receipt of any antibiotics, bismuth compounds, or proton pump inhibitors (PPI) within 1 year before the study and prior *H. pylori* eradication.
4. The presence of chronic renal failure, hepatic disease, congestive heart failure, or human immunodeficiency virus (HIV) infection.
5. Pregnancy or lactation.
6. Inability to follow up.

### Methods

All the patients were divided into three groups: ECA, RCA, and RCM (R: 20 mg rabeprazole, E: 40 mg esomeprazole, C: 0.5 g clarithromycin, A: 1.0 g amoxicillin, and M: 0.4 g metronidazole). PPI were administered before breakfast and dinner, and antibiotics were administered after breakfast and dinner. The patients were treated for 14 days and followed up for 1 year.

*H. pylori* was detected by the 14C-urea breath test (14C-UBT)^13^ using the 14C-urea breath machine (Shenzhen Haidewei Technology Co. Ltd., Shenzhen, China) before and 4 weeks after treatment. The cut-off value of the 14C-UBT was 100 dpm/mmol CO_2_. Clinical data were then collected.

The study was conducted at the First Affiliated Hospital of Guangxi University of Science and Technology, Liuzhou, China. The hospital is a tertiary care center. All experimental protocols were approved by the ethics and research committees of the First Affiliated Hospital of Guangxi University of Science and Technology. All methods were performed in accordance with the relevant guidelines and regulations from the Maastricht IV/Florence Consensus Report^14^. Informed consent was obtained from all the participants.

### Statistical analysis

Descriptive statistics were mean ± standard deviation (SD) for age and chi-squared or Fisher’s exact test for rates. Independent factors that may have been associated with successful eradication were assessed using multiple logistic regression analysis. The following variables were analyzed as independent factors: sex, age, lesion characteristics, and treatment groups. *p* values < 0.05 were thought to be statistically significant. All analyses were performed using IBM SPSS 19.0.

## Results

The clinical characteristics are presented in Table 1. The mean age of the patients was 44 ± 12 years (male: female, 3.2:1).

**Table 1:** Clinical characteristics of study subjects.

Clinical characteristics	Success (n)	Total (n)	Eradication rate (%)	*p* value
**Gender**				
Male	155	203	76	0.680
Female	45	61	74	
**Age**				
< 40 years	64	117	55	0.002
≥ 40 years	126	147	86	
**Lesion characteristics**				
Chronic gastritis	30	39	77	0.098
Gastric ulcers	27	45	60	Reference
Duodenal ulcers	102	129	79	0.012
Both gastric and duodenal ulcers	36	51	71	0.492
**Treatment groups**				
ECA	74	87	85	0.007
RCM	54	90	60	Reference
RCA	71	87	82	0.036

**Figure 1:**
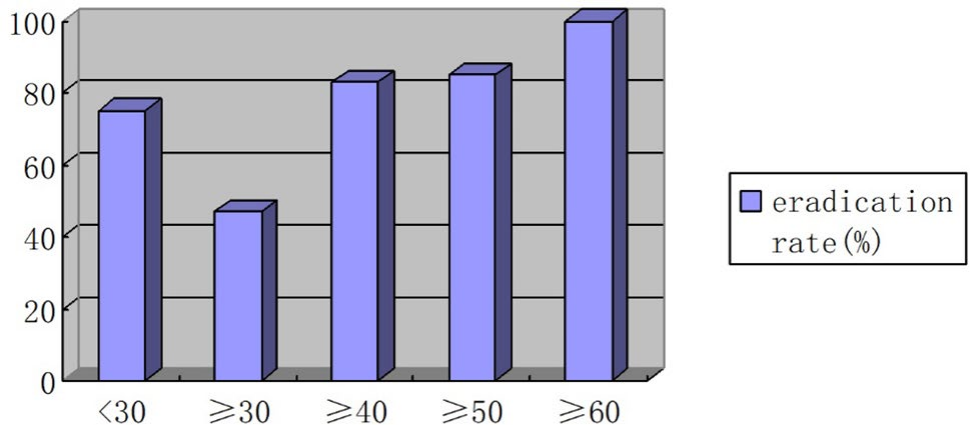
Eradication rates in the initial eradication therapy based on age group.

All eradication rates were analyzed per protocol because the patients who had completed the therapy were investigated. The eradication rates by age group were 75%, 47%, 83%, 85%, and 100% for < 30, 30–39, 40–49, 50–59, and ≥ 60-year-old patients, respectively. The eradication rate was the lowest in 30–39-year-old patients and the highest ≥ 60-year-old patients (47% vs. 100%, Fig. 1). The eradication rate in ≥ 40-year-old patients was higher than that in < 40-year-old patients, even after adjusting for other factors [odds ratio (OR) 4.58, *p* = 0.003, Table 2].

**Table 2:** Factors associated with *H. pylori* eradication.

Logistic regression model	Independent variable	Repeat 14C-UBT	Odds ratio in the final model (95% confidence interval)	*p* value in the final model*
1	Age ≥ 40 years	126/147 (86%)	4.58 (1.68–12.45)	0.003
	Age < 40 years	64/117 (55%)	1	
2	Male	155/203 (76%)	0.83 (0.23–2.97)	0.768
	Female	45/61 (74%)	1	

*Statistically significant *p* value < 0.05.

The eradication rates by lesion characteristics were 77%, 60%, 79%, and 71% for patients with chronic gastritis, gastric ulcers, duodenal ulcers, and both gastric and duodenal ulcers, respectively. The eradication rate in patients with duodenal ulcers was higher than that in patients with gastric ulcers (79% vs. 60%, *p* < 0.05). Moreover, the eradication rate was similar between males and females (76% vs. 74%, *p* = 0.68). The eradication rate did not differ significantly between the ECA and RCA groups (85% vs. 82%, *p* = 0.542). The eradication rates in the ECA (85%) and RCA (82%) groups were significantly higher than that in the RCM group (60%) (*p* = 0.007 and 0.036, respectively) (Table 1).

The incidence of adverse events was 9.5% (95% CI 0.7–18.3). Diarrhea and taste perversion were commonly observed. None of the patients discontinued the therapy because of adverse effects.

In the final logistic regression model, the effects of age on *H. pylori* eradication were estimated after adjusting for all other factors. The effects of lesion characteristics and treatment groups were not significant, and these factors were therefore not included in the final model.

## Discussion

Maastricht IV/Florence Consensus Report^14^ pointed out that different ways of improving the PPI-clarithromycin-amoxicillin/metronidazole regimens have been proposed such as increase of the dose of PPI and the length of treatment. Both the increase of the dose of PPI and extended duration of treatment have been considered in our study.

Our study revealed a link between age and *H. pylori* eradication therapy. Compared with < 40-year-old patients, we found a higher eradication rate in ≥ 40-year-old patients, even after adjusting for other factors. The eradication rate was 100% in patients aged over 60 years. As reported by Japanese scholars, patients aged under 50 years are prone to *H. pylori* eradication failure. The eradication rate in patients aged over 70 years has been found to be over 90%. Independent predictors of treatment success include older age^15,16^. The gastric mucosa is more atrophic in elderly patients than in younger patients and has hyposecretion of gastric acid. The ability of gastric acid to inactivate antibiotics decreases in elderly patients.

The incidence of PUD and bleeding complications is increasing in elderly patients worldwide. Approximately 53–73% of elderly patients with peptic ulcers are positive for *H. pylori*. The benefit of curing *H. pylori* infection in elderly patients with *H. pylori*-associated PUD and severe chronic gastritis has been demonstrated in a previous study^17^. The eradication rate in elderly patients was found to be higher in our study. Eradication should be performed in elderly patients.

There are several possible reasons for the lower eradication rate observed in young patients. One explanation is that young people tend to forget to take medicines because of busy work schedules^18^. Moreover, some younger patients stopped the therapy by themselves because their symptoms ameliorated shortly after taking PPI. Medication adherence and factors affecting adherence play a very important role in *H. pylori* eradication therapy. Another explanation is that young patients may have many bad habits, including irregular meal timings, smoking, alcohol abuse, and staying up too late. Finally, young patients may not have regular checkups.

It is particularly important to assess how the eradication outcomes can be improved in young patients. There is no simple solution to this problem. Several methods are recommended to resolve this issue. First, doctors should explain the necessity of eradication and the harmful consequences of irregular medication to patients. At the same time, bad habits, such as smoking, irregular meal timings, and alcohol abuse, should be stopped. Second, better therapeutic strategies and the reasons for eradication failure should be explored. Individualized treatment should be considered. Third, the follow-up program should be improved. Regular checkups should be recommended.

Previous studies have suggested that probiotic supplementation can increase the eradication rate in young patients^17,19^. A Japanese research team found that the use of a new antisecretory agent, potassium-competitive acid blocker (PCAB), instead of PPI and clarithromycin-based triple therapy increased the eradication rate in young to middle-aged patients^20^. Intragastric violet light phototherapy^21^ and bovine anti-*H. pylori* antibody-containing milk^22^ could eradicate *H. pylori*. Moreover, smoking cessation may increase the *H. pylori* eradication rate^23^. Correct evaluation of the quality improvement protocol can confirm the strategies that are most successful in every patient and dispel inaccurate perceptions, which can then be considered in the case of other patients as well.

We found that the eradication rate in patients with duodenal ulcers was significantly higher than that in patients with gastric ulcer (79% vs. 60%). A previous study revealed significantly different failure rates between patients with duodenal ulcers and non-ulcer dyspepsia (21.9% vs. 33.7%, *p* < 10^−6^)^24^. *H. pylori* therapy is always thought to be related to gastric acid secretion. Gastric acid secretion is low in patients with gastric ulcers and high in those with duodenal ulcers. The lower gastric acid concentration in patients with gastric ulcers to inactivate antibiotics decreases. So it’s difficult to explain the reason by the secretion of gastric acid. The reasons are not yet clearly understood. It could be related to patient differences, or the status of the gastric mucosa, or to differences in the infecting *H. pylori* strain. Multicenter clinical trials have indicated that patients with duodenal ulcers and non-ulcer dyspepsia should be managed differently in medical practice and considered independently in eradication trials^24^.

There are some limitations to our study. First, our study has a retrospective design. Many risk factors, such as habits (smoking and alcohol use), were not considered in our study. Second, our study was conducted at a single academic medical center and had sample bias. There are two advantages of conducting the study at our hospital. It is a large medical center that serves almost 5 million people. Moreover, patients with different occupations and from different places visit the hospital. Thus, patient diversity can be ensured. Third, the number of elderly patients was small. A larger sample size consisting of more elderly patients is needed for more careful investigation.

In conclusion, in our study, age was found to be associated with *H. pylori* eradication. Patients with duodenal ulcers had a higher *H. pylori* eradication rate than those with other lesions. Younger patients, especially those with gastric ulcers, had a lower eradication rate. Further studies are needed to explore why the eradication rate is lower and how it can be increased in these patients. Predicting the success of *H. pylori* eradication therapy and choosing the appropriate therapeutic schedule based on clinical parameters will benefit the patients and decrease healthcare costs.
